# Leptospirosis-Induced Septic Shock and Multi-Organ Dysfunction Syndrome: A Complex Case of Zoonotic Infection in a Young Female Patient

**DOI:** 10.7759/cureus.51243

**Published:** 2023-12-28

**Authors:** Nino Gvajaia, Mariam Tkeshelashvili, Levan Ratiani, Elene Pachkoria, Ia Mikadze

**Affiliations:** 1 Critical Care Medicine, Tbilisi State Medical University, American MD Program, Tbilisi, GEO; 2 Department of Anesthesiology and Reanimatology, Department of Infectious Diseases, The First University Clinic of Tbilisi State Medical University, Tbilisi, GEO

**Keywords:** zoonotic infections, leptospira interrogans, multidisciplinary approach, multiorgan failure, septic shock

## Abstract

Leptospirosis, a zoonotic infection caused by the *Leptospira* bacteria, can manifest with varying clinical severities, ranging from subclinical disease to severe multiorgan failure. This progression to severe multiorgan failure, also known as multi-organ dysfunction syndrome (MODS), is a life-threatening condition characterized by the dysfunction of two or more organ systems. Often, MODS is a consequence of events triggered by underlying pathologies, such as severe infections, including those caused by *Leptospira*. Here, we present a case report of a 29-year-old female patient who initially sought care for increased temperature, fatigue, diarrhea, and vomiting. The patient exhibited signs of septic shock (SS). Her medical history raised suspicion of multiple potential sources of infection. She experienced cat scratch several days before admission, accompanied by an enlarged inguinal lymph node and a history of frequent interaction with animals, as well as freshwater exposure, which prompted investigations into various zoonotic infections. Empiric treatment was started, and, subsequently, after several days, *Leptospira* titer came back positive. Over the hospitalization course, the patient developed multi-organ failure, which was attributed to the underlying *Leptospira* infection. The complexity and severity of the patient's condition underscore the far-reaching impact of leptospirosis in precipitating a spectrum of systemic complications culminating in multiorgan failure. The treatment interventions yielded successful outcome, and the patient recovered in one month. This case report highlights the difficulties in diagnosing and treating patients with multiple possible sources of infection. it emphasizes the need for a careful history-taking and high level of suspicion for zoonotic infections in patients with a history of animal exposure and clinical symptoms suggestive of infectious diseases.

## Introduction

Sepsis remains a significant reason for admission to intensive care units (ICUs) and is a leading cause of mortality [1]. Because it is a time-sensitive emergency, it is crucial to have early and effective therapeutic measures to improve patient survival rate [1]. The underlying reason for septic shock (SS) in our patient was leptospirosis, which presents a diagnostic challenge due to its diverse clinical spectrum, ranging from flu-like symptoms to a swiftly fatal infection. Its differential diagnosis is notably complex, given the overlap in clinical manifestations that often mimic other infectious diseases, such as brucellosis, dengue fever, and malaria [2].

The transmission and clinical characteristics of leptospirosis vary based on different socioeconomic and environmental conditions. It exhibits a higher prevalence in the tropical regions of lower-income countries, whereas in developed nations, this disease frequently receives inadequate attention [2]. Recently, the prevalence of leptospirosis in Georgia has increased, which can be attributed to advancements in the diagnosis of the infection and the emergence of new serogroups of *Leptospira*, which are the outcome of the country's intense migration process [3].

## Case presentation

A 29-year-old female patient came to the hospital due to a three-day history of general weakness, nausea, and fever. On the day before admission, the patient had visited a different clinic where a computed tomography (CT) scan revealed a 29 mm nonhomogeneous nodule with clear boundaries in the subcutaneous tissue of the left groin. The surgeon evaluated the nodule and found no need for immediate surgery, as the lab results and abdominal CT scan were normal. The patient got recommendations for antibiotic and antipyretic therapy and was discharged.

On the next day, the patient's temperature increased to 39°C, and she remained unresponsive to antipyretic medication. In addition, she experienced diarrhea and vomiting, with the vomitus displaying a green color. She was admitted to our hospital. At that time, the patient had a normal neurological status. Abdominal examination revealed mild diffuse tenderness on palpation. An enlarged, painful lymph node in the left groin area extending to the front of the thigh was observed. She said that the pain started three days ago before admission to our hospital. It was constant, with 8 out of 10 in severity, and she described it as sharp and nonradiating. She reported that movements were making the pain worse. It was also discovered that the patient had a cat at home and that she had experienced a small, superficial cat scratch on the forearm two days before admission. Given the patient's recent history of recreational activities in an endemic region involving freshwater exposure and her frequent close interactions with animals, clinical suspicion for leptospirosis arose, and a microscopic agglutination test (MAT) was requested for *Leptospira*.

Upon admission, the patient displayed signs and symptoms of sepsis. Her vital signs were as follows: pulse rate (PR) of 120 beats per minute, blood pressure of 75/45 mmHg, respiratory rate (RR) of 30 breaths per minute, oxygen saturation (SpO_2_) of 87%, and skin temperature of 39°C.

The patient's condition deteriorated over the next two days, and adequate oxygenation was not achieved due to worsening acute hypoxemic respiratory failure, leading to the necessity of mechanical ventilation. On the following week, the case was complicated by multi-organ dysfunction syndrome (MODS), indicated by the development of respiratory failure and mixed cardiogenic and SS, abnormal liver function tests (LFTs), and the detection of effusion in the peritoneal and pleural cavities. The patient developed pneumonia, and consolidation was seen on radiological findings. Moreover, the patient's condition progressed to acute kidney failure, heart failure, and infective endocarditis (IE).

Diagnostic evaluation

Diagnostic testing began following initial routine care in the emergency and ICU. On the first day of hospitalization, CBC showed normocytic anemia, mild thrombocytopenia, and leukocytosis (with neutrophilia). Electrolyte check revealed severe hypokalemia. However, arterial blood gas (ABG) analysis revealed no significant acid-base balance disturbance. In addition, procalcitonin (PCT) evaluation showed a level of 24.8 ng/ml and serum C-reactive protein level of 207 mg/L. The creatinine level was significantly increased to 243 mmol/L. A coagulation panel was also performed, and the results revealed elevated PT and aPTT. Concurrently, a notable decrease in the fibrinogen level to 1.2 g/L was observed, reflecting significant alterations in the coagulation profile. A biopsy of the lesion could not be performed due to the risk of bleeding. Blood culture was found to be sterile.

Upon admission, samples were sent for several zoonotic infections, including *Leptospira*, *Brucella,* and *Bartonella*. The MAT exhibited a fourfold rise in *Leptospira* antibody titers only, indicative of a substantial increase in specific agglutination, suggesting an active or recent infection with the *Leptospira* bacteria. It was confirmed by repeated testing conducted on two separate occasions. As the patient was admitted with three days of severe diarrhea and vomiting, stool studies were also obtained, which revealed the presence of *Clostridioides difficile *toxins A and B. 

Two days after being on mechanical ventilation, bacteriological examination of respiratory secretions showed *Klebsiella pneumoniae,* and antibiogram revealed resistance to all antibiotics except colistin (polymyxin E). 

Throughout the initial two weeks of the patient’s hospital stay, several imaging tests were conducted. To assess the patient’s respiratory distress, a bedside chest X-ray was also taken, which showed bilateral diffuse opacities throughout both lung fields with air bronchograms that was suggestive of adult acute respiratory distress syndrome. The results of a bedside echocardiogram revealed multiple, echogenic mobile masses attached to valvular structures with mitral and aortic regurgitation, as well as slightly decreased ejection fraction (45%). 

Management plan and follow-up

Besides supportive therapy, initial antibiotic treatment included piperacillin/tazobactam IV 4.5 grams every eight hours with enoxaparin subcutaneous injection as a part of sepsis and SS management protocol. Furthermore, based on the patient's clinical features and history, empiric therapy was started with doxycycline 100 mg every 12 hours.

The patient’s condition started to improve after two weeks. She has been under sedation for 10 days, with periodic dosage reductions that the patient accepted better over time. On the 16th day of hospitalization, follow-up echocardiography showed that the ejection fraction returned to 55%, and the degree of mitral regurgitation also significantly decreased.

**Figure 1:**
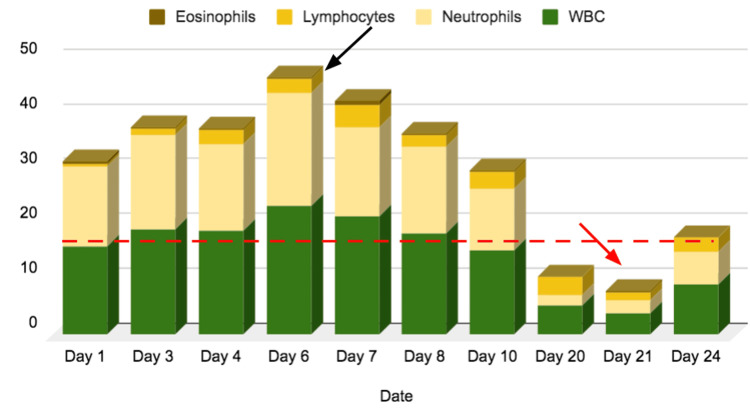
CBC changes throughout the hospitalization period

WBC counts with differentials were acquired during the patient’s hospital stay, as shown in Figure 1. On day 6 of the hospital stay, the WBC count peaked (black arrow), but it began declining steadily. On day 21 of the hospital stay (red arrow), the highest decline in the WBC count with differentials was observed, consistent with the patient’s clinical condition (the dashed red line indicates the upper limit of the WBC normal value range). During the hospital stay, the platelet level also changed. Platelet levels increased after two weeks in the ICU. 

**Figure 2:**
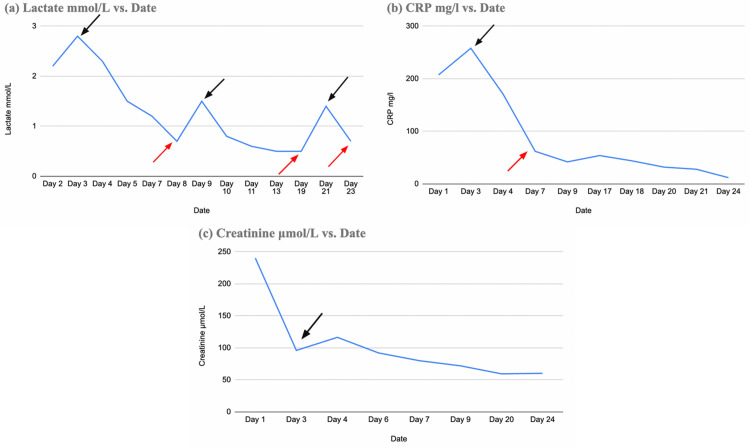
Lactate, C-reactive protein, and creatinine levels during the hospitalization period

Throughout the patient’s hospital stay, the lactate level changed, but pH readings remained within the normal range. It had several peak values, but the overall course yielded a successful decline (Figure 2A) and was already within the normal range when the patient was discharged.

C-reactive protein (CRP) levels fluctuated during the patient’s hospital stay. The CRP level peaked on day three of hospitalization (black arrow, Figure 2B), and on day four (red arrow, Figure 2B), it gradually decreased, indicating a favorable clinical prognosis. The patient’s creatinine level was markedly low on day three of hospitalization (black arrow, Figure 2C), indicating the restoration of end-organ function and a favorable clinical outlook.

A second blood culture was performed two weeks following the hospital stay, and as it was seen on the previous test, the results were sterile. PCT levels gradually returned to normal, indicating a favorable clinical prognosis.

**Figure 3:**
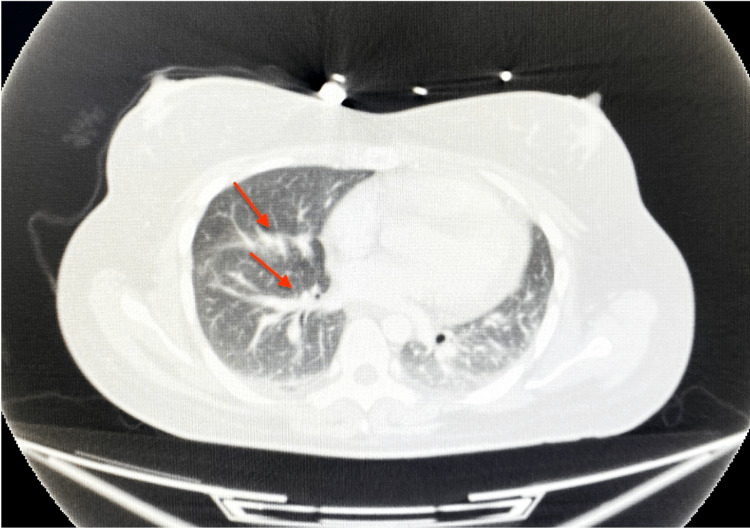
Chest CT (computed tomography) scan

On the 16th day after ICU admission, chest CT was performed, and no signs of thrombosis were seen. The fluid in the pericardium was within normal limits. Figure 3 shows that the transparency in both lungs was reduced, and infiltration areas were visible in the lower lobes (red arrows). There was a small effusion in the bilateral pleural cavity, and no air was seen.

Overall, the patient’s condition significantly improved. On the 20th day after ICU admission, the patient was extubated after her respiratory failure reversed. In the left inguinal region, lymphadenopathy disappeared on palpation. On the 27th day of admission, the patient was transferred from the intensive care unit to a general ward and was discharged after two weeks.

## Discussion

The discussion will delve into critical aspects of the case, including the role of diagnostic evaluations and the importance of early diagnosis and interdisciplinary care for improving patient outcomes.

As early diagnosis allows for the timely initiation of appropriate interventions, it is crucial in managing multiorgan failure [4]. When evaluating the potential causes of sepsis in patients presenting with positive systemic inflammatory response syndrome (SIRS) criteria, it is essential to consider leptospirosis as a possible etiology, particularly in locations where the disease is endemic [5]. Leptospirosis is a bacterial infection caused by the *Leptospira* genus, which can affect multiple organ systems in the body. In regions where leptospirosis is prevalent, people can become infected through contact with water (e.g., from swimming, wading, kayaking, or fishing) that has been contaminated with the urine of infected animals. Immediate initiation of appropriate antibiotic treatment and supportive care plays a crucial role in saving the lives of these patients [6].

In this case, the diagnostic challenge was caused by the patient's initial presentation with nonspecific symptoms and potential multiple sources of infection, for example, cat scratch, frequent animal contact, recent history of freshwater exposure, and an inguinal mass. As confirmed later, the patient had a positive antibody titer for *Leptospira*. As this infection has a broad range of clinical manifestations, it is often misdiagnosed, or diagnosis is delayed [6].

In this patient, the development of severe SS and MODS is indicative of the severe form of leptospirosis. Leptospirosis can lead to a systemic inflammatory response, causing a widespread endothelial damage, which can manifest as acute kidney injury, liver dysfunction (as evidenced by elevated liver function tests in this patient), IE, and possibly meningitis.

Concurrently, the utilization of PCT, a marker for host response and blood infection, has garnered significant interest and has already been endorsed for guiding antimicrobial therapy in patients suffering from sepsis [7]. PCT offers valuable supplementary information that can complement clinical and diagnostic indicators. As a result, it has a profound impact on treatment decisions for patients suspected of having infections or sepsis and the duration of antibiotic treatment courses [7]. Furthermore, CRP serves as a sensitive marker in cases of sepsis, indicating the presence of acute infection or inflammation. The serial monitoring of its level offers the opportunity to adjust the duration of antibiotic therapy, shortening it once CRP levels return to normal, while also enabling the early identification of patients who may not be responding well to treatment [8]. Another potential biomarker besides CRP and PCT is MCP-1, a soluble chemokine secreted by various cells under pro-inflammatory conditions in sepsis. Elevated levels of MCP-1 in sepsis patients have been associated with organ dysfunction and may indicate a poor prognosis [9].

Generally, SS constitutes a significant risk factor for mortality in various medical conditions, including IE, which entails a poorer prognosis. In most studies, the total mortality ranges from 20% to 25%. Currently, up to 40-50% of affected patients need valve surgery at some point in the clinical course, with the overall mortality staying at 20-25% per year in most reported studies [10]. IE, diagnosed by echocardiogram, exacerbated the patient's presentation as it induced mitral regurgitation and altered valve function [10]. 

All the other infections present in this patient, for example, *Klebsiella pneumonia*, is likely ventilator-associated, and *C. difficile* is due to antimicrobial treatment. The presence of a subcutaneous nodule in the patient's groin area, which later developed into hyperemia of the skin, could represent the site of entry for *Leptospira*. As *Bartonella* also presents with self-limiting lymphadenopathy in the draining site of cat scratch [11], this infection was also suspected, but serological analysis revealed negative results.

In this case, the patient received antibiotic therapy based on the results of bacterial cultures and antibiotic sensitivity testing. Numerous studies investigating sepsis and SS have consistently demonstrated that delayed administration of antibiotics is associated with unfavorable outcomes. Improving the efficacy of antimicrobial therapy while limiting the emergence of resistance strains is a primary concern in managing patients with sepsis and SS. Joseph and Rodvold defined the optimal antimicrobial therapy using the concept of the four D's: "right drug, right dose, de-escalation, and right duration." This approach highlights the key principles for effective and appropriate use of antimicrobial agents in treating infectious diseases [12].

The treatment of severe leptospirosis includes appropriate antibiotics and supportive management. Piperacillin/tazobactam and doxicycline was used for seven days with supportive care in this patient. She recovered in a period of one month. When a patient presents with unspecific signs of infection, leptospirosis should be considered as one of the possible etiology [6].

## Conclusions

In conclusion, this case report illustrates a severe and complex presentation of leptospirosis in a 29-year-old female, characterized by the rapid progression to septic shock and MODS. The patient's clinical journey underscores the challenges in diagnosing and managing leptospirosis, a zoonotic disease with a diverse clinical spectrum. The diagnosis was complicated by overlapping symptoms with other infectious diseases and further exacerbated by secondary hospital-acquired infections, including multi-drug resistant *K. pneumoniae *and *C. difficile* colitis.

The case report raises awareness of potential complications associated with multi-organ failure. Serial measurements of inflammatory markers, organ function tests, and imaging studies played a vital role in monitoring the patient's response to treatment and guiding therapeutic decisions. The insights gained from this report contribute to the existing literature and serve as a reminder of the importance of early recognition, comprehensive evaluations, and multidisciplinary collaboration in managing these complex cases.
